# Determining the mechanisms underlying augmented renal drug clearance in the critically ill: use of exogenous marker compounds

**DOI:** 10.1186/s13054-014-0657-z

**Published:** 2014-11-29

**Authors:** Andrew A Udy, Paul Jarrett, Janine Stuart, Melissa Lassig-Smith, Therese Starr, Rachel Dunlop, Steven C Wallis, Jason A Roberts, Jeffrey Lipman

**Affiliations:** Department of Intensive Care and Hyperbaric Medicine, The Alfred, 55 Commercial Road, Prahran, Melbourne, Victoria 3181 Australia; Burns, Trauma, and Critical Care Research Centre, The University of Queensland, Herston, Brisbane, Queensland 4029 Australia; Department of Intensive Care Medicine, Royal Brisbane and Women’s Hospital, Butterfield Street, Herston, Brisbane, Queensland 4029 Australia

## Abstract

**Introduction:**

The aim of this study was to explore changes in glomerular filtration (GFR) and renal tubular function in critically ill patients at risk of augmented renal clearance (ARC), using exogenous marker compounds.

**Methods:**

This prospective, observational pharmacokinetic (PK) study was performed in a university-affiliated, tertiary-level, adult intensive care unit (ICU). Patients aged less than or equal to 60 years, manifesting a systemic inflammatory response, with an expected ICU length of stay more than 24 hours, no evidence of acute renal impairment (plasma creatinine concentration <120 μmol/L) and no history of chronic kidney disease or renal replacement therapy were eligible for inclusion. The following study markers were administered concurrently: sinistrin 2,500 mg (Inutest; Laevosan, Linz, Austria), p-aminohippuric acid (PAH) 440 mg (4% p-aminohippuric acid sodium salt; CFM Oskar Tropitzsch, Marktredwitz, Germany), *rac*-pindolol 5 or 15 mg (Barbloc; Alphapharm, Millers Point, NSW, Australia) and fluconazole 100 mg (Diflucan; Pfizer Australia Pty Ltd, West Ryde, NSW, Australia). Plasma concentrations were then measured at 5, 10, 15, 30, 60 and 120 minutes and 4, 6, 12 and 24 hours post-administration. Non-compartmental PK analysis was used to quantify GFR, tubular secretion and tubular reabsorption.

**Results:**

Twenty patients were included in the study. Marker administration was well tolerated, with no adverse events reported. Sinistrin clearance as a marker of GFR was significantly elevated (mean, 180 (95% confidence interval (CI), 141 to 219) ml/min) and correlated well with creatinine clearance (*r* =0.70, *P* <0.01). Net tubular secretion of PAH, a marker of tubular anion secretion, was also elevated (mean, 428 (95% CI, 306 to 550) ml/min), as was net tubular reabsorption of fluconazole (mean, 135 (95% CI, 100 to 169) ml/min). Net tubular secretion of (*S*)- and (*R*)-pinodolol, a marker of tubular cation secretion, was impaired.

**Conclusions:**

In critically ill patients at risk of ARC, significant alterations in glomerular filtration, renal tubular secretion and tubular reabsorption are apparent. This has implications for accurate dosing of renally eliminated drugs.

## Introduction

The kidneys have a crucial role in water and electrolyte homeostasis, acid–base balance, excretion of nitrogenous waste products and select endocrine functions. In terms of drug disposition, renal excretion of parent compounds or active metabolites represents a key route of drug elimination for many agents. This process involves a combination of glomerular filtration, renal tubular secretion and renal tubular reabsorption. Net renal drug elimination represents the sum of these processes.

Recent research has highlighted that renal antibacterial drug elimination (particularly renally cleared β-lactams) can be significantly elevated in the critically ill [1], such that drug exposure is often suboptimal [1-3], and may lead to adverse clinical outcomes [4]. This phenomenon has been termed augmented renal clearance (ARC) [1], and is seen in association with an elevated urinary creatinine clearance (CL_CR_) [5]. Such measures have therefore been advocated as a useful tool to identify ARC in the critically ill [6,7], the assumption being that elevated glomerular filtration is a key mechanism in this process.

However, measured CL_CR_ suffers from the limitations inherent to using any endogenous filtration marker, such that variations in diet, muscle mass and physical activity may also influence plasma CR concentrations. In addition, CL_CR_ provides limited mechanistic information on changes in renal tubular function. As such, renal drug handling in the critically ill requires additional research, ideally using exogenous indicators of renal function. One method involves the administration of multiple renal markers [8], such that the elimination of each can be used to quantify changes in glomerular filtration, and tubular drug handling, simultaneously. Using such a ‘cocktail’, Gross and colleagues applied this approach in 12 healthy male subjects [9], establishing useful baseline values for comparison.

The aims of this prospective observational study were therefore (1) to assess the feasibility and safety of administering multiple markers of renal function simultaneously in the intensive care unit (ICU), (2) to quantify glomerular filtration and renal tubular function in a cohort of critically ill patients at risk of ARC and (3) to compare these data with previously reported values in healthy volunteers.

## Material and methods

### Setting

This single-centre observational study was undertaken in a tertiary-level, university-affiliated ICU. Ethical approval was obtained from our institution’s Human Research Ethics Committee (HREC/09/QRBW/15), with written informed consent obtained from either the patient or the nominated substitute decision-maker.

### Study population

Study participants had to be aged ≤60 years, have an expected ICU length of stay >24 hours, evidence of a systemic inflammatory response syndrome [10] in the 24 hours prior to marker administration, a plasma CR concentration <120 μmol/L and no history of chronic kidney disease (CKD) or renal replacement therapy. Patients were excluded from receiving any study markers if (1) either invasive haemodynamic monitoring or an indwelling urinary catheter (IDC) was not employed as part of standard management; (2) they were <18 years of age; (3) they were pregnant; (4) rhabdomyolysis was clinically suspected or the plasma creatinine kinase level was >5,000 IU/L; (5) they were in the ‘risk’ category or greater for acute kidney injury, as defined by the risk, injury, failure, loss, and end-stage (RIFLE) kidney disease criteria [11]; (6) there was a documented allergy and/or contraindication to one or more of the renal markers; (7) one or more markers were being employed clinically; or (8) the treating clinician considered the patient unsuitable for enrolment. Recruitment was carried out by convenience sampling.

### Data collection, dosing administration and sampling protocol

Demographic data, including age, sex, height, weight, Acute Physiology and Chronic Health Evaluation (APACHE) II score and admission diagnosis were recorded prospectively. After confirming eligibility and obtaining consent, the following study markers were administered; sinistrin (Inutest; Laevosan, Linz, Austria) 2,500 mg in 10 ml intravenously (IV) over 30 seconds, 4% p-aminohippuric acid sodium salt (PAH; CFM Oskar Tropitzsch, Marktredwitz, Germany) 440 mg IV over 1 minute, *rac*-pindolol (Barbloc; Alphapharm, Millers Point, NSW, Australia) 5 or 15 mg orally or via an enteral feeding tube and fluconazole (Diflucan; Pfizer Australia Pty Ltd, West Ryde, NSW, Australia) 100 mg orally or via an enteral feeding tube.

Enteral markers were administered only if the patient was documented to be tolerant of enteral nutrition. *rac*-Pindolol was withheld if there was (1) a history of severe reactive airway disease (asthma and/or chronic obstructive airway disease) or symptomatic bradyarrhythmia or (2) moderate vasopressor support (>10 μg/min infusion of noradrenaline or adrenaline) was being provided. Administration of all or a combination of markers was possible, depending on the patient profile.

Blood samples were then taken via the intra-arterial cannula at the following time points after marker administration: 5, 10, 15, 30, 60 and 120 minutes and 4, 6, 12 and 24 hours. Following collection, all blood samples were immediately placed on ice and centrifuged within 60 minutes at 3,000 rpm for 10 minutes. Plasma was then aliquoted off and stored for analysis at −80°C. All urine was collected via the IDC over the same 24-hour period, with a 10-ml sample stored at −80°C for later assays. The remaining urine was forwarded to the local hospital laboratory for biochemical analysis.

All marker administration was performed under medical supervision, with the patients receiving continuous cardiovascular and respiratory monitoring. All physiological parameters, fluid balance and any therapeutic interventions performed were recorded concurrently.

### Bioanalysis

(*S*)-pindolol, (*R*)-pindolol, PAH and fluconazole in plasma and urine were measured by in-house liquid chromatography-tandem mass spectrometry methods validated in accordance with US Food and Drug Administration guidelines for bioanalysis. Separations were tailored for each analyte: a reverse-phase column was used for fluconazole; a hydrophilic interaction liquid chromatography column was used for PAH; and a chiral column was used for pindolol. Sinistrin was measured by using a commercially available enzyme-linked immunosorbent assay kit (FIT-GFR; BioPAL, Worcester, MA, USA). CR was measured in plasma and urine by using an isotope dilution mass spectrometry traceable assay through the institutional pathology laboratory. For comparison, estimated GFR (eGFR) was calculated using the Chronic Kidney Disease Epidemiology Collaboration (CKD-EPI) formula [12].

### Data treatment and statistical analysis

CL_CR_ was determined from the urinary CR concentration, 24-hour urine volume and plasma CR concentrations obtained from routine clinical testing. If multiple plasma values were available over the 24-hour study period, the mean was used in subsequent analysis. Plasma concentrations of all renal markers were plotted on a concentration/time graph, with the slope of the terminal portion calculated by linear regression. Suitability for use in further analysis was assessed by visual inspection. Backward extrapolation to time zero was performed for those markers administered IV (sinistrin and PAH). The area under the plasma concentration/time curve (AUC_0-∞_) was determined using the linear trapezoidal rule and extrapolated to infinity by adding the product of the last measured plasma concentration multiplied by the terminal slope. As per previous recommendations, plasma concentrations to at least 120 minutes were required for accurate calculation of sinistrin AUC_0-∞_ [13]. Plasma CL for each marker was then calculated as dose/AUC_0-∞_.

The total amount recovered in urine (Ae) of PAH, fluconazole and (*S*)- and (*R*)-pindolol was calculated by multiplying the urinary concentration by the 24-hour urine volume. Renal clearance (CL_R_) was then calculated as Ae/AUC_0-∞_, and non-renal clearance (CL_NR_) as the difference of CL − CL_R_. Sinistrin CL was taken as representing the GFR, and PAH CL_R_ was taken as effective renal plasma flow (ERPF). The filtration fraction was calculated as GFR/ERPF. Glomerular filtration of the unbound marker was calculated as *f*_u_ × GFR, where *f*_u_ is the unbound fraction in plasma. Tubular anion secretion was determined from the net tubular secretion of PAH as CL_R_ − (*f*_u_ × GFR). Tubular cation secretion was determined from the net tubular secretion of (*S*)- and (*R*)-pindolol as CL_R_ − (*f*_u_ × GFR). Tubular reabsorption was estimated from the net reabsorption of fluconazole as (*f*_u_ × GFR) − CL_R_ [8]. The *f*_u_ of PAH was taken as 0.83 [9]; for (*S*)- and (*R*)-pindolol, it was 0.45 [14]; and for fluconazole, it was 0.83 [9].

Continuous data are presented as the mean (95% confidence interval (CI)). Categorical data are presented as counts (%). For bivariate correlation between continuous variables, we used a Pearson correlation coefficient (*r*). A paired Student’s *t*-test was used to compare intra-patient data. Bland-Altman analysis was employed to explore the agreement between sinistrin CL, CL_CR_ and CKD-EPI eGFR values. A double-sided *P*-value <0.05 was considered as statistically significant, and SPSS version 22 software (IBM, Armonk, NY, USA) was used for all analyses.

## Results

### Demographic, illness severity and physiological data

Twenty adult patients were recruited into the study. Demographic, illness severity, therapeutic and physiological data are provided in Table 1. As presented, the subjects were mostly male, young and primarily admitted after trauma. As per the inclusion criteria, plasma CR concentrations were low; the mean was 64.5 (53.2 to 75.7) μmol/L. Twenty-four-hour CL_CR_ measures were elevated (168 (139 to 197) ml/min), whereas in comparison CKD-EPI eGFR estimates were significantly lower (128 (113 to 144) ml/min, *P* <0.01).

**Table 1:** **Demographic, illness severity and physiological data** ^**a**^

**Variable**	**Data (** ***N*** **=20)**
Age, yr, mean (95% CI)	36.7 (29.2 to 44.1)
Males, *n* (%)	12 (60.0)
Height, m, mean (95% CI)	1.74 (1.68 to 1.79)
Weight, kg, mean (95% CI)	80.2 (70.8 to 89.5)
Body surface area, m^2^, mean (95% CI)	1.94 (1.81 to 2.07)
Days in ICU before sampling, mean (95% CI)	4.90 (3.74 to 6.06)
APACHE II score, mean (95% CI)	17.3 (13.7 to 20.8)
Diagnosis, *n* (%)	
Burns	3 (15.0)
Trauma (including head injury)	10 (50.0)
Neurological	3 (15.0)
Sepsis	3 (15.0)
Other	1 (5.0)
Mechanically ventilated, *n* (%)	19 (95.0)
Receiving vasopressor infusion, *n* (%)	3 (15.0)
Pre-dose HR, beats/min, mean (95% CI)	100 (90.8 to 110)
Pre-dose MAP, mmHg, mean (95% CI)	89.4 (83.0 to 95.8)
Plasma CR, μmol/L, mean (95% CI)	64.5 (53.2 to 75.7)
Urinary CR, mmol/L, mean (95% CI)	5.93 (4.02 to 7.85)
24-hr urinary volume, L, mean (95% CI)	3.10 (2.35 to 3.85)
Urinary pH, mean (95% CI), *n* =16	6.16 (5.75 to 6.57)
24-hr fluid balance, ml, mean (95% CI)	700 (−126 to 1,527)
24-hr CL_CR_, ml/min, mean (95% CI)	168 (139 to 197)
CKD-EPI eGFR, ml/min, mean (95% CI)	128 (113 to 144)

^a^APACHE, Acute Physiology and Chronic Health Evaluation; CI, Confidence interval; CL_CR_, Creatinine clearance; CKD-EPI, Chronic Kidney Disease Epidemiology Collaboration; CR, Creatinine; eGFR, Estimated glomerular filtration rate; HR, Heart rate; ICU, Intensive care unit; MAP, Mean arterial pressure.

### Pharmacokinetic analyses of individual markers

**Figure 1:**
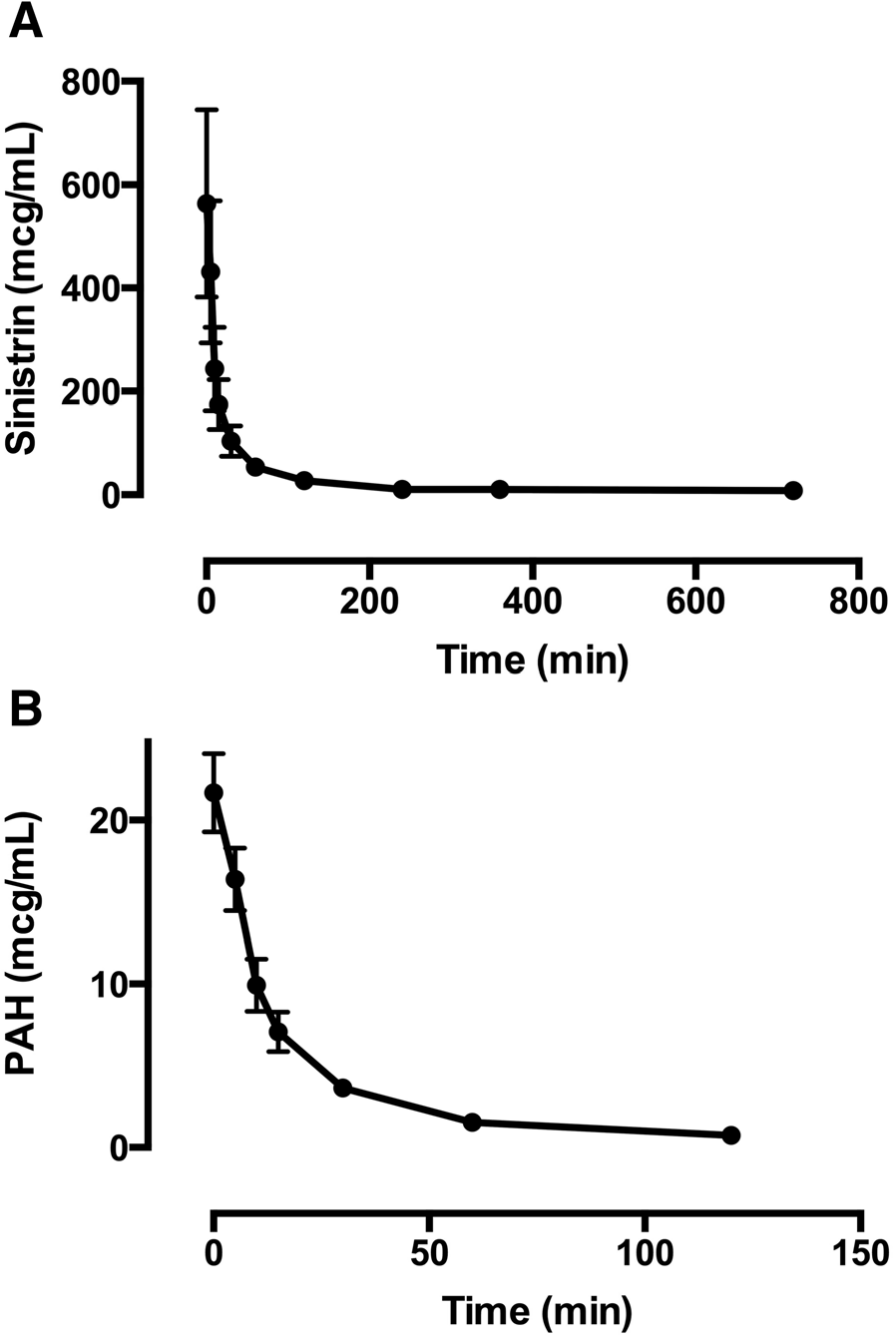
**Plot of plasma concentration versus time for intravenous study markers** ***.*** Mean (95% confidence interval) plasma sinistrin **(A)** and p-aminohippuric acid (PAH) **(B)** concentrations (*y*-axis) versus time (*x*-axis) are shown.

**Figure 2:**
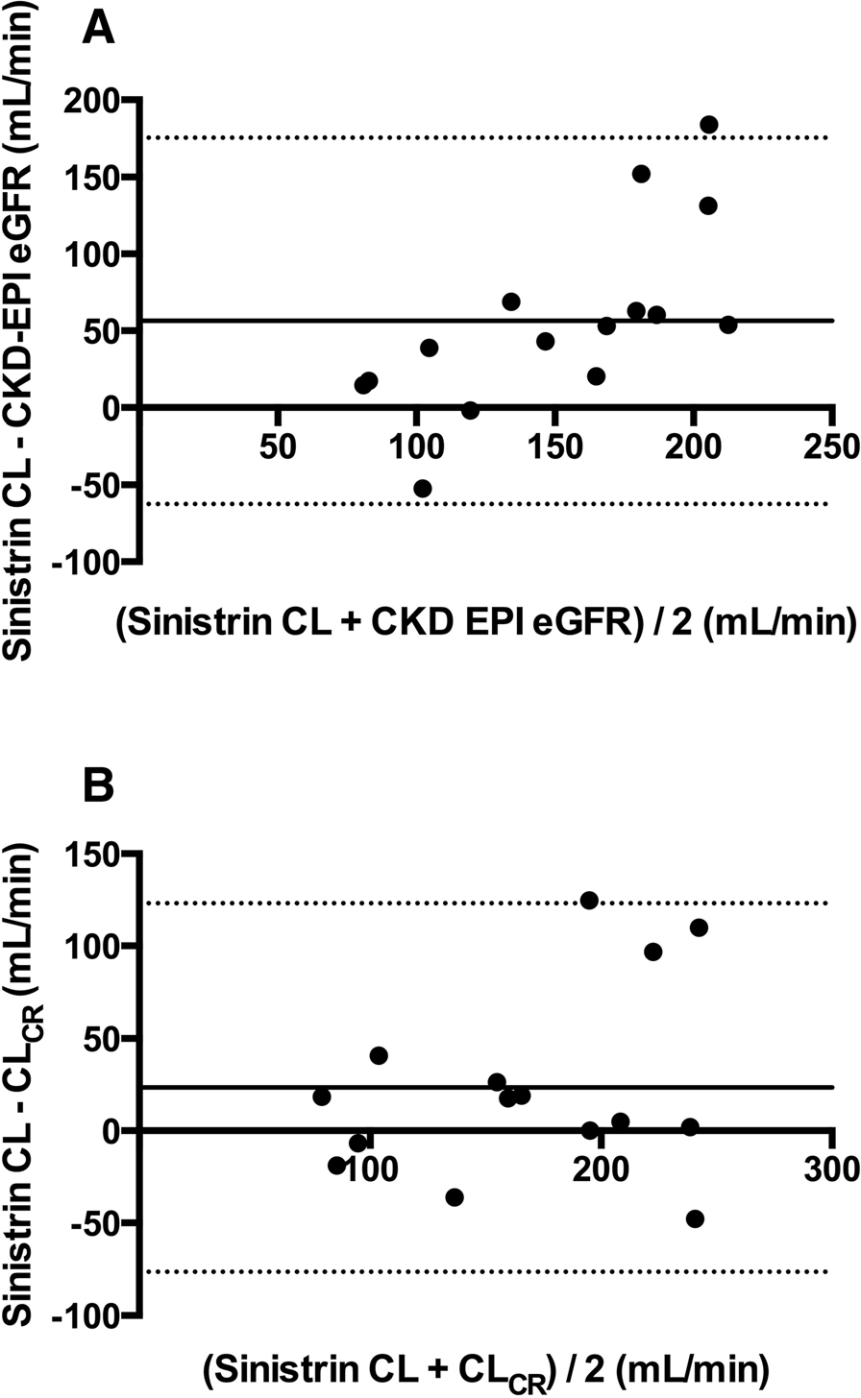
**Bland-Altman plots of sinistrin clearance versus creatinine clearance and Chronic Kidney Disease Epidemiology Collaboration estimated glomerular filtration rate.** Comparison of the difference between sinistrin clearance (CL) and Chronic Kidney Disease Epidemiology Collaboration estimated glomerular filtration rate (CKD-EPI eGFR) **(A)** and creatinine clearance (CL_CR_) **(B)** on the *y*-axis versus the average value on the *x*-axis. The solid lines represent the bias (mean difference), and the dotted lines represent the 95% limits of agreement.

**Figure 3:**
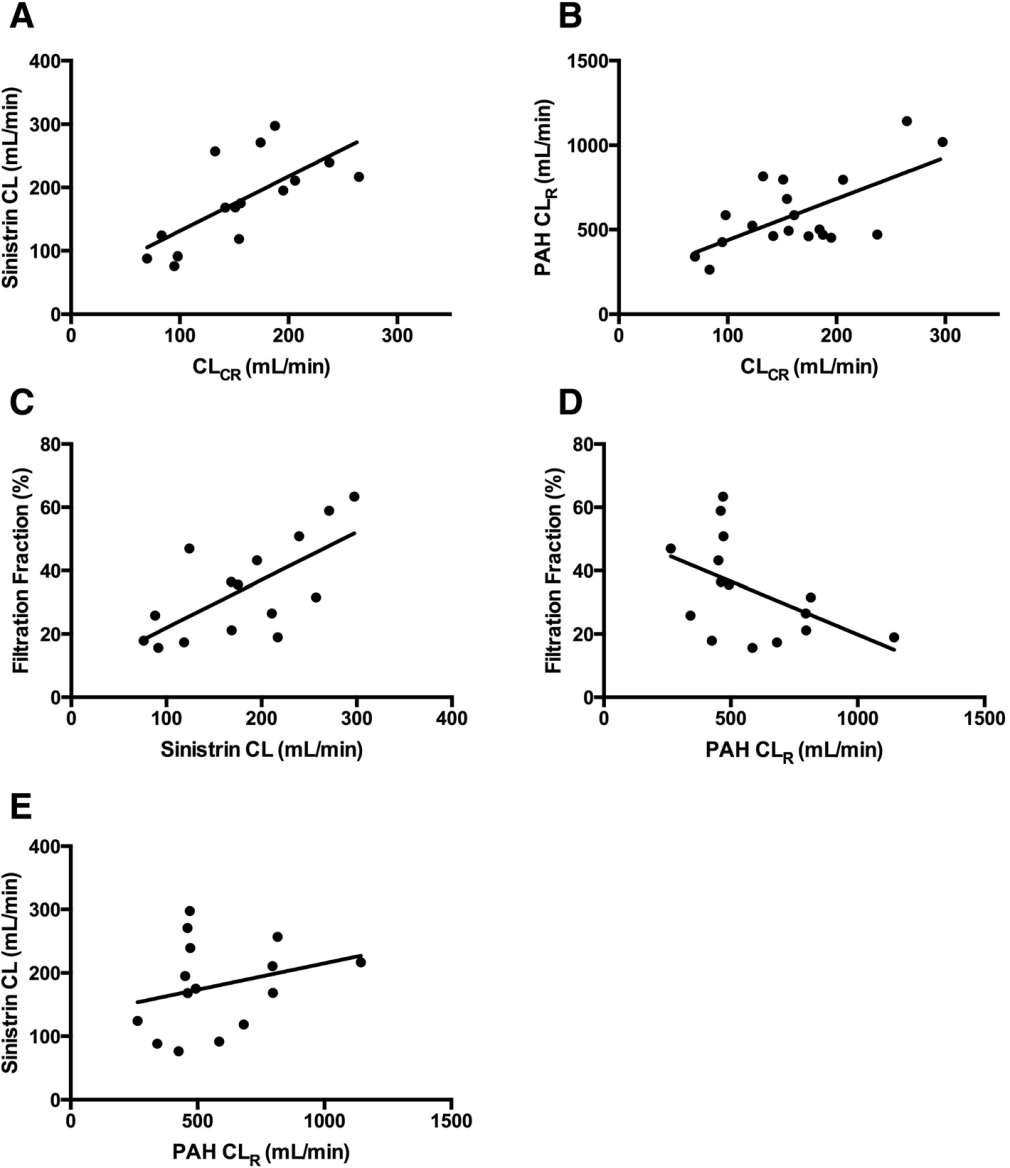
**Graphical correlations between sinistrin clearance, creatinine clearance, p-aminohippuric acid renal clearance and filtration fraction. (A)** Sinistrin clearance (CL) (*y*-axis) versus creatinine clearance (CL_CR_) (*x*-axis) (*r* =0.70, *P* <0.01). **(B)** p-Aminohippuric acid renal clearance (PAH CL_R_) (*y*-axis) versus CL_CR_ (*x*-axis) (*r* =0.65, *P* <0.01). **(C)** Filtration fraction (*y*-axis) versus sinistrin CL (*x*-axis) (*r* =0.68, *P* <0.01). **(D)** Filtration fraction (*y*-axis) versus PAH CL_R_ (*x*-axis) (*r* = −0.49, *P* =0.06). **(E)** Sinistrin CL (*y*-axis) versus PAH CL_R_ (*x*-axis) (*r* =0.27, *P* =0.32). A linear regression line has been fitted in each case.

Sinistrin and PAH were administered IV to all 20 patients, with no adverse effects observed. Suitable sinistrin plasma concentration/time profiles could be plotted in 15 cases, which are presented in Figure 1. PAH concentrations were assessable in 19 cases, and these are also illustrated graphically in Figure 1. Both sinistrin CL and PAH CL_R_ were elevated, although moderate inter-patient variation in these parameters was observed (Table 2). The mean filtration fraction was 34.0% (25.4% to 42.7%). Of note, sinistrin CL was higher than CL_CR_, although this was not statistically significant (*P* =0.10). These measures were highly correlated, however (*r* =0.70, *P* <0.01). Bland-Altman plots comparing sinistrin CL with CL_CR_, as well as with CKD-EPI eGFR, are presented in Figure 2. Graphical correlations between sinistin CL, CL_CR_, PAH CL_R_ and filtration fraction are presented in Figure 3.

**Table 2:** **Pharmacokinetic analyses of individual markers** ^**a**^

	**Sinistrin**	**PAH**	**(** ***S*** **)-pindolol**	**(** ***R*** **)-pindolol**	**Fluconazole**
	**(** ***n*** **=15)**	**(** ***n*** **=19)**	**(** ***n*** **=14)**	**(** ***n*** **=14)**	**(** ***n*** **=18)**
AUC_0-∞_, mcg/ml/min, mean (95% CI)	16,513 (12,247 to 20,778)	426 (366 to 487)	8.70 (3.07 to 14.3)	11.2 (1.42 to 21.0)	3,804 (3,073 to 4,535)
CL, ml/min, mean (95% CI)	180 (141 to 219)	1,139 (943 to 1,335)	654 (465 to 842)	639 (393 to 884)	30.5 (24.4 to 36.6)
Ae, mg, mean (95% CI)	–	230 (211 to 249)	0.80 (0.36 to 1.24)	0.77 (0.39 to 1.15)	35.9 (27.4 to 44.3)
CL_R_, ml/min, mean (95% CI)	–	594 (484 to 704)	122 (77 to 167)	98 (68 to 129)	12.3 (7.61 to 16.9)
CL_NR_, ml/min, mean (95% CI)	–	545 (439 to 652)	532 (369 to 695)	541 (310 to 772)	18.2 (15.5 to 21.0)
CL_NR_, mean (95% CI)	–	47.8 (43.5 to 52.0)	80.9 (75.8 to 86.0)	81.5 (76.3 to 86.8)	64.1 (55.7 to 72.6)
CL_CR_, ml/min, mean (95% CI)	156 (125 to 188)	164 (135 to 193)	166 (127 to 204)	166 (127 to 204)	171 (140 to 203)

^a^Ae, Amount recovered in urine; AUC_0-∞_; Area under the concentration/time curve (extrapolated to infinity); CI, Confidence interval; CL, Clearance; CL_CR_, Creatinine clearance; CL_NR_, Non-renal clearance; CL_R_, Renal clearance; PAH, p-Aminohippuric acid.

**Figure 4:**
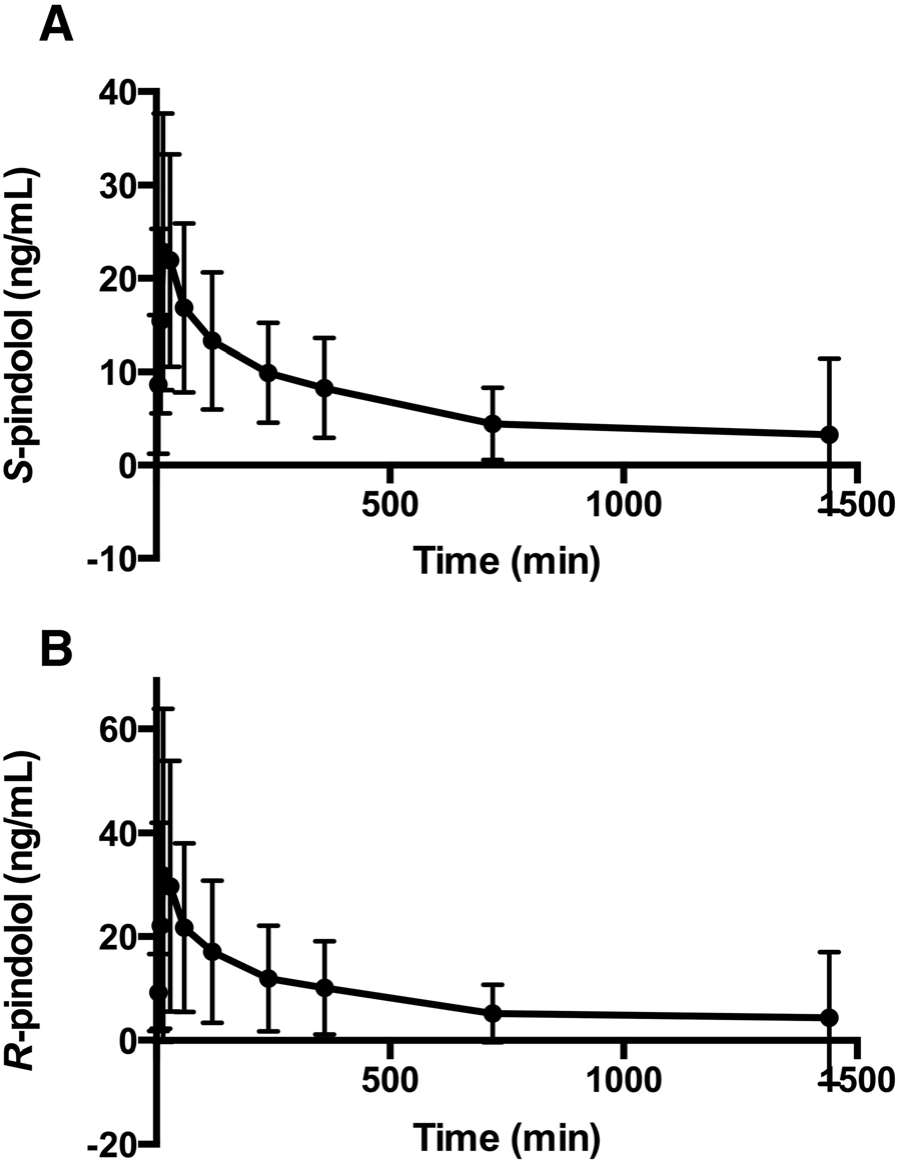
**Plot of plasma concentration versus time for (** ***S*** **)- and (** ***R*** **)-pindolol** ***.*** Mean (95% confidence interval) plasma (*S*)-pindolol **(A)** and (*R*)-pindolol **(B)** concentrations (*y*-axis) versus time (*x*-axis) are plotted.

**Figure 5:**
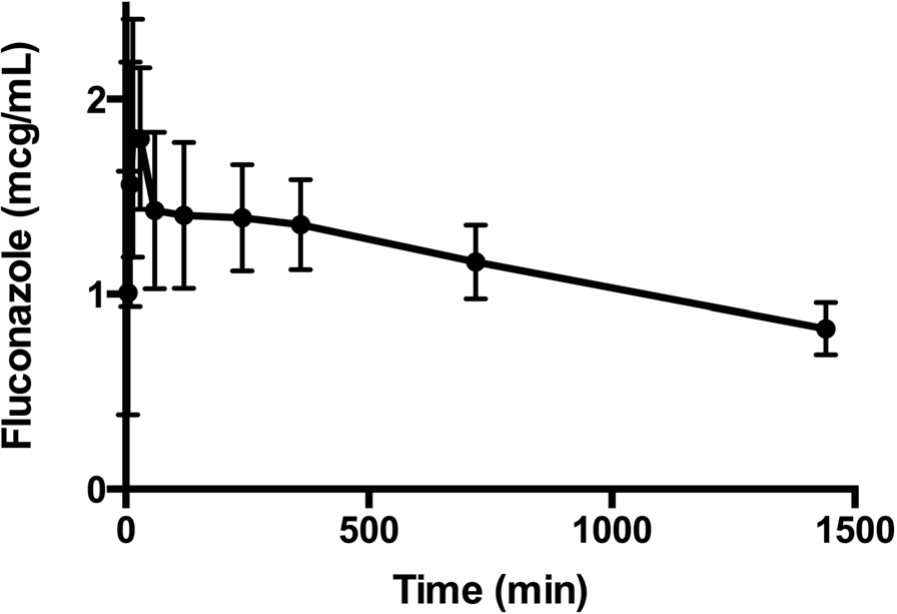
**Fluconazole plasma concentration versus time plot** ***.*** Mean (95% confidence interval) plasma fluconazole concentration (*y*-axis) versus time (*x*-axis) values are plotted.

*rac*-Pindolol was administered enterally to 17 patients and fluconazole to 19, without any observed adverse events. Suitable concentration/time profiles could be plotted in 14 cases for (*S*)- and (*R*)-pindolol and in 18 cases for fluconazole. These are presented in Figures 4 and 5, respectively. Ae, AUC, CL, CL_R_ and CL_NR_ values for each marker are presented in Table 2.

### Glomerular and tubular functional assessment and combined marker analyses

GFR, ERPF, net tubular anion secretion, net tubular cation secretion and net tubular reabsorption were calculated as outlined above. These data, in comparison to those previously reported in healthy volunteers, are presented in Table 3.

**Table 3:** **Glomerular and tubular functional assessment and combined marker analyses** ^**a**^

	**Mean (95% CI) (present study)**		**Healthy volunteers** ^**b**^ **mean (SD)**
	**ml/min/1.73 m** ^**2**^	**ml/min**	**ml/min**
Glomerular filtration rate (*n* =15)	161 (129 to 193)	180 (141 to 219)	143 (45)
Effective renal plasma flow (*n* =19)	535 (442 to 628)	594 (484 to 704)	467 (146)
Renal tubular anion secretion (*n* =15)			
Net secretion of PAH	388 (282 to 494)	428 (306 to 550)	359 (134)
Renal tubular cation secretion (*n* =10)			
Net tubular secretion of (*S*)-pindolol	47 (−19 to 113)	46 (−15 to 107)	152 (70)
Net tubular secretion of (*R*)-pindolol	25 (−16 to 67)	24 (−17 to 66)	132 (45)
Renal tubular reabsorption (*n* =13)			
Net tubular reabsorption of fluconazole	120 (92 to 148)	135 (100 to 169)	93 (29)

^a^CI, Confidence interval; PAH, p-Aminohippuric acid; SD, Standard deviation. ^b^Twelve healthy male subjects (mean age, 24 years; mean weight, 72 kg; mean height, 1.78 m) with normal laboratory parameters and a Cockcroft-Gault estimated creatinine clearance >100 ml/min [9].

## Discussion

In comparison with previous data reported in healthy volunteers, by using identical exogenous markers of renal function, we have demonstrated elevated glomerular filtration and renal tubular anion secretion in a selected cohort of critically ill patients at risk of ARC. As these mechanisms are central to β-lactam renal elimination, the potential for inadequate drug exposure is considerable in this setting [7], an assertion consistent with recent literature [2-4,15].

Overall, the study protocol was well tolerated by all participants, with no adverse events observed. Furthermore, suitable concentration/time profiles could be generated in most instances, limited primarily by our strict criteria for determining sinistrin AUC_0-∞_ [13]. Sinistrin CL in our study was higher (see Table 3) than previously reported in non–critically ill subjects [9,16,17]. In keeping with this, CL_CR_ measures were also elevated, with good correlation (*r* =0.7) between these values.

PAH CL was also higher in our cohort, although mechanisms other than renal excretion of unchanged drug accounted for a significant fraction. This process reflects acetylation of PAH [18], which has previously been reported to account for 15% to 30% of total drug elimination [18-20]. Fluconazole CL in the critically ill has been reported to be between 0.88 to 2.17 L/hr [21]. The findings of the present study are consistent with these previous reports, although fluconazole CL_NR_ was significantly elevated in our study. This metabolic pathway involves glucuronide conjugation in the liver, a process that appears to decline in parallel with deteriorating renal function [22]. Of note, total CL and CL_R_ of both enantiomers of pindolol were lower than previously reported in healthy volunteers [14].

The reduced CL_R_ of *rac*-pindolol and the suggestion of impaired renal cation secretion were unexpected findings. A likely explanation may involve variation in urinary pH, as previously described by Ujhelyi *et al*. [23]. In their study, (*S*)- and (*R*)-pindolol CL_R_ was determined in eight healthy male subjects before and after urinary acidification with ammonium chloride. At a mean urinary pH of 5.0, a twofold increase in CL_R_ of both isomers was noted [23] and thought to be principally related to an increase in organic transporter activity. In our study, the mean (95% CI) urinary pH was 6.16 (5.75 to 6.57), considerably more alkaline than might have been expected and which may account for the reduced CL_R_ observed.

Another potential mechanism impairing *rac*-pindolol tubular secretion may involve inhibition of the organic cation transport system, as has been demonstrated with co-administration of cimetidine [24]. Of note, this is thought primarily to involve competitive inhibition of the luminal efflux process rather than basolateral uptake [25]. Although none of the study participants were concurrently receiving cimetidine, other medications have been potentially implicated [26], resulting in varying degrees of inhibition. As such, whereas co-administration of the study markers themselves do not generate any interactions [9,27,28], a potential additional drug–drug interaction in our study cohort cannot be excluded.

The observed changes in CL_NR_ of both PAH and fluconazole are notable findings. Acetylation of PAH occurs in the liver and kidneys, with the metabolite (*N*-acetyl-PAH) then excreted in urine [29]. In contrast to previous work [18-20], plasma PAH CL was significantly elevated in our study, with CL_NR_ accounting for, on average, 48% of total PAH elimination. Similarly, fluconazole CL_NR_ was also elevated, a finding disparate from prior research in the critically ill [30]. This may be a consequence of greater tubular reabsorption of fluconazole, a process driven by increased glomerular filtration. Notwithstanding this, as non-renal elimination of PAH and fluconazole primarily involve conjugation, these observations remain consistent with increased solute delivery to other drug eliminating organs, such as the liver. Whether augmented hepatic clearance also occurs in this setting remains uncertain, although, given the potentially significant impact of such changes on PK parameters, this represents an essential area for future study.

Elevated CL_CR_ has been reported in numerous subgroups, including traumatic brain injury [31], polytrauma [32,33], burns [34], sepsis [35], ventilator-associated pneumonia [36], meningitis [37] and major surgery [38]. Lower illness severity scores [39], younger age [35], male sex [4] and systemic inflammation [40] are common features. Indeed, young male patients admitted post-trauma and without the requirement for vasopressor therapy formed the majority of our study cohort. Activation of renal reserve represents a possible explanation for these findings [41], although data from our study reinforce that changes in GFR in this context are likely more complex. Specifically, for a given ERPF, the filtration fraction varied considerably, accounting for the increased sinistrin CL observed in some patients (Figure 3). These data indicate that changes in intra-glomerular filtration pressure (due to variable afferent and efferent arteriolar tone) are potentially a key mechanism warranting further in-depth study.

CKD-EPI eGFR values were significantly lower than CL_CR_ measures, reinforcing the limited utility of these estimates in such patients [42]. CL_CR_ measures displayed less bias in comparison to sinistin CL, although precision was poor with either estimate (Figure 2). Nonetheless, low-normal plasma CR concentrations in young trauma patients without oliguria should alert the clinician to the possibility of ARC, following which a urinary CL_CR_ test could be used to reinforce this finding. Of note, the use of CL_CR_ as a surrogate outside a population at risk of ARC remains uncertain, particularly given the increasing inaccuracy of this measure with declining renal function [43].

We wish to declare the following limitations. We chose to employ study markers previously validated in healthy volunteers, allowing for direct comparison with a non–critically ill cohort. Our use of enteral pindolol and fluconazole mirror the approach used by Gross *et al*. [9], although gastrointestinal absorption in the critically ill is often more variable. Importantly, both fluconazole [44] and pindolol [45] have excellent oral bioavailability, and none of the study participants receiving these markers were intolerant of enteral nutrition.

Our sampling interval was also limited to 24 hours, as opposed to longer periods that have been used to characterize fluconazole pharmacokinetics [46]. However, fluconazole CL_R_ over this shorter period is comparable with that based on sampling for 120 hours [9], allowing for a shorter study period. Finally, we chose to measure ERPF by using a PAH bolus IV approach rather than constant infusion. Although there has been some criticism of this technique [47], peak concentrations were substantially lower than those known to saturate anion transport in the kidney [19], such that our analyses are likely to provide a reliable measure of PAH CL_R_.

## Conclusions

In this study, we employed multiple exogenous markers to quantify renal elimination pathways in patients at risk of ARC. Increased sinistrin CL (in correlation with a high CL_CR_) confirms elevated glomerular filtration in these patients. In addition, increases in tubular anion secretion and tubular reabsorption were noted, which may also influence renal elimination for drugs subject to these clearance mechanisms. Tubular cation secretion was impaired, although the reason for this observation remains uncertain. Non-renal elimination of PAH and fluconazole were also increased, a finding that warrants further investigation.

## Key messages

- In patients at risk of ARC, sinistrin clearance is elevated, an observation consistent with increased glomerular filtration.
- Sinistrin clearance is moderately correlated with measured creatinine clearance in these patients.
- Increased renal tubular anion secretion and renal tubular reabsorption were also noted in this group.
- Renal tubular cation secretion was impaired, possibly a reflection of changes in urinary pH.
- Non-renal elimination mechanism were also elevated, a finding that warrants further investigation.

## Abbreviations

Ae: Amount recovered in urine
APACHE: Acute Physiology and Chronic Health Evaluation
ARC: Augmented renal clearance
AUC_0-∞_: Area under the concentration/time curve extrapolated to infinity
CI: Confidence interval
CKD: Chronic kidney disease
CKD-EPI: Chronic Kidney Disease Epidemiology Collaboration
CL: Plasma clearance
CL_CR_: Creatinine clearance
CL_NR_: Non-renal clearance
CL_R_: Renal clearance
CR: Creatinine
ERPF: Effective renal plasma flow
*f*_u_: Unbound fraction
GFR: Glomerular filtration rate
ICU: Intensive care unit
IDC: Indwelling urinary catheter
IV: Intravenous
PAH: p-Aminohippuric acid
PK: Pharmacokinetic
RIFLE: Risk, injury, failure, loss, end-stage kidney disease criteria
SD: Standard deviation
